# The dilemma of cytomegalovirus and hepatitis B virus interaction

**DOI:** 10.1093/gastro/goac018

**Published:** 2022-05-26

**Authors:** Muzammil M Khan, Mukarram J Ali, Hira Hanif, Muhammad H Maqsood, Imama Ahmad, Javier E G Alvarez, Maria-Andreea Catana, Daryl T Y Lau

**Affiliations:** 1 Department of Medicine, Division of Gastroenterology, Liver Research Center, Beth Israel Deaconess Medical Center, Harvard Medical School, Boston, MA, USA; 2 Department of Medicine, North Shore Medical Center, Salem, MA, USA

**Keywords:** CMV, HBV, HBV CMV co-infection, CMV reactivation, HBV reactivatio, n, IgG Avidity test

## Abstract

Hepatitis B virus (HBV) remains a global public health problem despite the availability of effective vaccine and antiviral therapy. Cytomegalovirus (CMV), another hepatotropic virus, is also very prevalent in the general population worldwide. Both HBV and CMV can persist in the host and have potential to reactivate especially with weakened host cellular immunity. Superimposed CMV infection can lead to severe HBV reactivation. The pathogenesis of the co-infection of HBV and CMV remains poorly understood. Studies reported conflicting results regarding the inhibitory effect of CMV on HBV replication. There is an unmet need on the management of co-infection of HBV and CMV; research initiatives dedicated to understanding their interactions are urgently needed.

Core tip

- CMV superimposed on HBV infection can have debilitating effects. Guidelines need to be established to provide standardized management especially among the immunocompromised patients. For patients with reactivation of previously stable chronic hepatitis B, acute CMV coinfection needs to be considered with timely evaluation and treatment.

## Introduction

Hepatitis B virus (HBV) is a significant public health challenge with ∼240 million infections worldwide [1]. Patients with chronic hepatitis B (CHB) usually have minimal symptoms and remain undiagnosed for years leading to cirrhosis, liver failure, and hepatocellular carcinoma [2]. For those with spontaneous recovery from acute hepatitis B, loss of immunologic control is still possible and can lead to active HBV replication. Reactivation of HBV can be spontaneous or triggered by immunosuppressed states including organ transplant and human immunodeficiency virus infection. Co-infection with other hepatotropic viruses such as cytomegalovirus (CMV), Epstein-Barr virus (EBV), and hepatitis E virus (HEV) can lead to an acute flare-up of CHB [3, 4]. CMV is a member of the herpesviridae and can persist in the host in a latent state after primary infection. Both CMV and HBV are prevalent and have the potential to persist and reactivate; the co-infection and interaction of these two viruses especially during the immunocompromised state can be challenging to diagnose and manage. In this review, we discuss the impact of CMV in the setting of HBV co-infection.

## HBV

### Epidemiology and natural history

**Figure 1.**
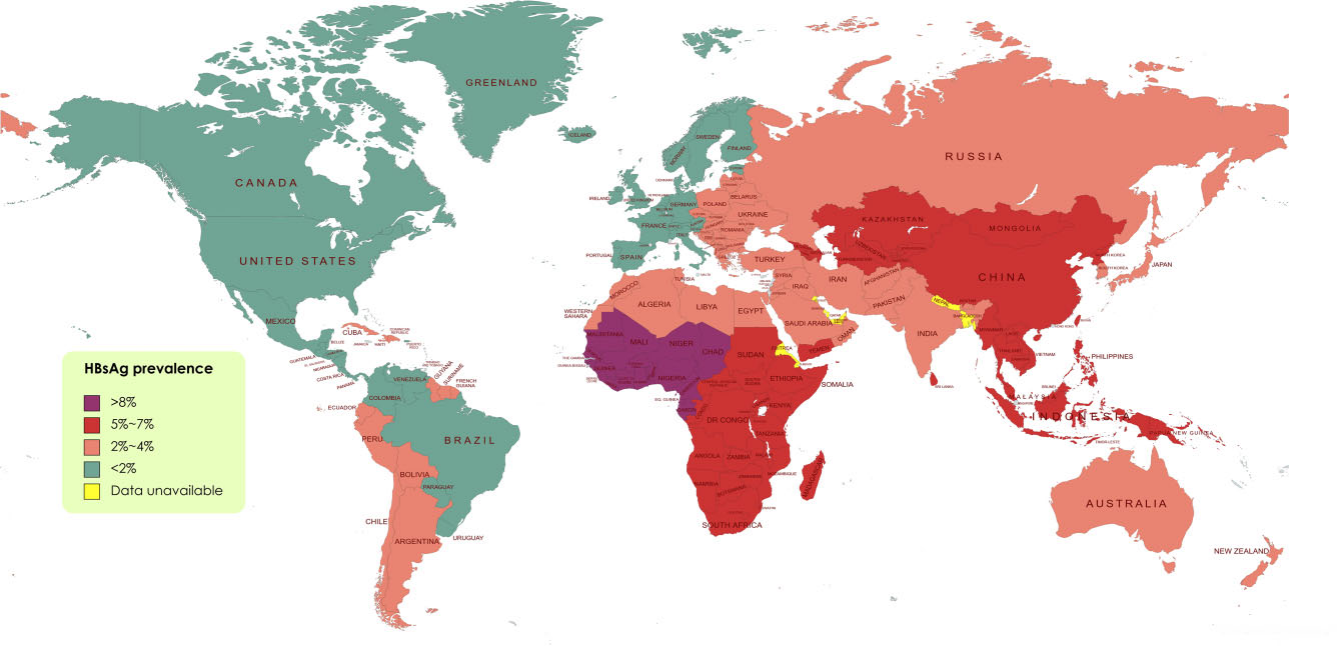
Global endemicity of hepatitis B virus. Source: Center for Disease Control and Prevention, CDC 2010.

It is estimated that ∼250–340 million people are infected with HBV, corresponding to a prevalence of 3.9% (3.4%–4.6%) of the world populations in 2016 [5]. CHB can progress to cirrhosis, liver failure, hepatocellular carcinoma, and death [6]. The distribution of HBV varies widely in different geographical regions (Figure 1). The high endemic regions are the Western Pacific and African regions (9.8%–10%) followed by Southeast Asia (6.8%), whereas the lowest prevalence is noted in the North and South American regions (0.3%) [5]. HBV can be transmitted via blood or sexual contact, and exposure during infancy by vertical transmission or during early childhood by horizontal transmission [7].

### HBV reactivation

The definitions of HBV reactivation are variable. The American Association for the Study of Liver Diseases (AASLD) defines it as (i) HBV DNA elevation compared with baseline, or an absolute increase in HBV DNA level if baseline is not available; and (ii) seroconversion to hepatitis B surface antigen (HBsAg) (+) from HBsAg (–), HBcAb(+) persons [8]. The Asian Pacific Association for the Study of Liver defined HBV reactivation as (i) a new appearance of HBV DNA level of ≥100 IU/mL or >2 log increase from baseline levels in a person with previously stable or undetectable levels; or (ii) detection of HBV DNA level of 20,000 IU/mL if baseline is unavailable [9]. It is important to emphasize that HBV reactivation can occur even among those who have recovered from hepatitis B with nonreactive HBsAg.

Host, viral, and immunosuppression are the key factors that increase the risk of HBV reactivation [10]. Host factors include male sex, older age, pregnancy, progression of human immunodeficiency virus (HIV) infection, and underlying diseases that require immunosuppressive therapies [10–13]. Viral factors are high HBV DNA levels, HBeAg positivity, HBV genotype, and HBsAg mutations [11]. Compared with the patients with HBsAg (–)/anti-HBc (+), the patients with HBsAg (+) are eight times more likely to have HBV reactivation [14]. HBV reactivation also depends on the type and degree of immunosuppressive therapies used. Immunosuppressive regimens have been categorized as high-risk (>10% risk), moderate-risk (1%–10% risk) or low-risk (<1% risk) based on their ability to cause reactivation [8]. The high-risk group includes anti-CD20 monoclonal antibodies and hematopoietic stem-cell transplantation with or without graft-vs-host disease; the moderate-risk group includes corticosteroids (prednisolone ≥20 mg for ≥4 weeks), anthracyclines (i.e. doxorubicin, epirubicin), and tyrosine kinase inhibitors (i.e. imatinib); and the low-risk group consists of corticosteroids for ≤4 weeks and immunosuppressive monotherapy such as methotrexate or azathioprine [10, 15].

Besides immunosuppressive drugs, the progressive immunodeficiency that accompanies chronic infection with HIV can also result in HBV reactivation and HBsAg seroeversion among patients with anti-HBc (+)/HBsAg (–) previously [16–18]. Many antiretroviral agents used in HIV also have activity against HBV including Lamivudine, Tenofovir, and Emtricitabine, and withdrawal of these drugs has been related to hepatitis B flare [19]. HBV reactivation has also been reported after discontinuation of antiviral therapy in immunocompetent individuals with chronic hepatitis B [20].

Recently, the reactivation of HBV in patients with HBV–hepatitis C virus (HCV) co-infection after the eradication of HCV with direct-acting antiviral agents (DAA) has been reported [21, 22]. The incidence of HBV reactivation ranged from 0.3% to 21.1% depending on the baseline HBsAg status [21, 23]. In a large retrospective study on veterans, only 9 of 62,290 (0.014%) HCV patients treated with DAA had HBV reactivation [22]. In that study, reactivation was defined as an increase of >1,000 IU/mL in HBV DNA or HBsAg detection in patients with negative HBsAg previously; HBsAg positivity prior to DAA therapy was associated with the greatest risk of HBV reactivation [22].

Superinfection with other viruses can also lead to significant reactivation of HBV in immunocompetent patients. Aslam *et al.* [4] reported a case of acute HEV leading to HBV reactivation in a 39-year-old Vietnamese woman who had stable HBeAg-negative CHB presenting with non-specific symptoms of fatigue and mild right-upper-quadrant tenderness.

CMV has a direct cytopathic effect on the hepatocytes and can lead to severe hepatitis. It is often under-diagnosed in immunocompetent individuals, especially if they have other underlying viral hepatitis [24]. We recently encountered such a case of a 73-year-old Asian male with well-controlled chronic hepatitis B who developed acute hepatitis secondary to cytomegalovirus superinfection. For the past 10 years, he was on tenofovir disoproxil fumarate (TDF) with optimally suppressed HBV DNA and minimal hepatic fibrosis. He presented to the emergency department with progressive fatigue, nausea, right-sided abdominal pain, dark urine, and scleral icterus for ∼6 weeks, and was noted to have elevated serum aminotransferase. He had a recent history of noncompliance with antiviral therapy so the initial working diagnosis was acute HBV breakthrough due to interruption of antiviral therapy. HBV DNA, however, was only minimally detectable. Upon further inquiry, the patient mentioned that he experienced symptoms even prior to TDF interruption. Other potential causes of acute liver disease were screened. The anti-CMV IgM returned positive. CMV treatment was not initiated as his jaundice and liver tests had improved. After 3 months, the anti-CMV IgM became nonreactive.

Due to the masquerading nature of superinfection with hepatotropic viruses, they are often overlooked in the setting of chronic hepatitis B [25]. Medication noncompliance is a common cause of CHB exacerbation; this case illustrated the importance of taking a detailed history to rule out all possible etiologies such as CMV, a dormant hepatotropic virus. In order to deal with such cases, we have presented an algorithmic approach that can be incorporated in our daily practice to manage patients in a better way (Figure 3).

Acute CMV infection or acute infection with other viruses such as hepatitis A, E, D, and C, and EBV can cause HBV exacerbation and can lead to fulminant hepatic failure and death [26]. Stransky *et al.* [27] identified CMV reactivation or superinfection as a cause of acute exacerbation of chronic hepatitis B in 8% of the patients.

## CMV

### Epidemiology and natural history

CMV infection has a seroprevalence varying from 40% to 100% in immunocompetent adults globally. Significant variations exist depending on the age and socio-economic status with a high prevalence almost approaching 100% in developing countries and generally a low prevalence in Western Europe and the USA [28, 29]. A recent meta-analysis by Zuhair *et al.* [30] reported that the global prevalence of CMV was ∼83%; the rates were highest between 88% and 90% in Eastern Mediterranean, Western Pacific, and African regions. The rate was lowest at 66% (95% uncertainty interval, 56%–74%) in European regions. According to the National Health and Nutrition Examination Survey (NHANES) 1999–2004 data in the USA, the age-adjusted CMV prevalence was 50.4%, which increased with age. The seroprevalence of CMV of those age 6–11 years was 37.5%; it reached almost 58% by age 40–49 years [31]. Female sex, foreign birthplace, high household crowding, and low household education and income were independent factors associated with higher CMV seroprevalence.

CMV can be transmitted to healthy individuals via contaminated body fluids including urine, stool, genital secretions, blood, saliva, and breast milk, or direct contact with contaminated environmental surfaces [32–34]. Compared with adults and older children, children aged 1–2 years shed the virus more frequently; they serve as the key transmitters of the virus [32]. Vertical transmission of CMV from mother to child is the most common cause of congenital infection accounting for 0.7–4.5 million cases per year in developing countries and 0.12 million cases annually in developed countries [34]. The majority of CMV transmission to the fetus occurs during primary infection of the pregnant women and less frequently with CMV reinfection or reactivation [34, 35].

The severity of CMV infection is determined by the state of the host immune system. Healthy adults are usually asymptomatic during acute infection but they may experience mononucleosis-like symptoms [36, 37]. CMV can establish latency in the endothelial cells, smooth muscle cells, fibroblasts, and epithelial cells via hematogenous dissemination that is primarily mediated by infected peripheral monocytes [38, 39]. This latent infection is generally kept in check by the cellular immunity driven by the T-cells. However, diminished cell-mediated immunity in the immunocompromised state can lead to severe CMV reactivation [40].

### Serology of primary CMV infection and reactivation

**Figure 2.**
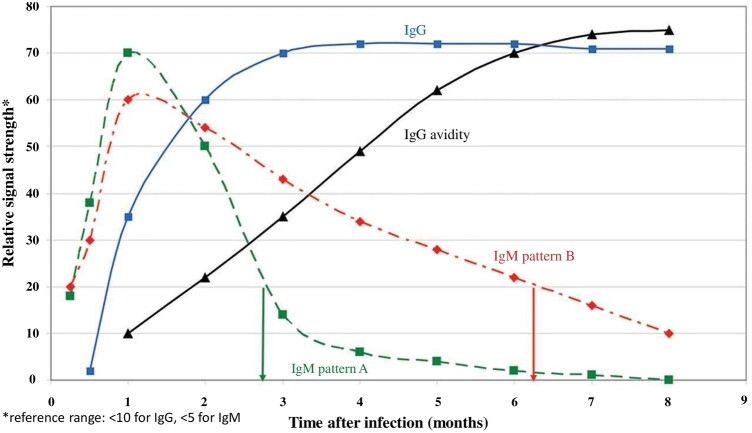
Serological markers of cytomegalovirus (CMV). Serological course of CMV antibodies. IgM (primary infection) appears 2–4 weeks after the contamination and stays in the blood for 3–6 months depending on the immune system of the host. Serum IgG indicates previous exposure or infection. CMV IgG avidity distinguishes primary infection from reactivation [41].

The acute and prior CMV infection can be diagnosed by measuring the IgG and IgM antibody response against CMV. During the initial acute infection, the CMV-specific IgM antibodies usually appear early within 2–4 weeks of infection, which can persist for ≤6 months. The CMV-specific IgG antibodies emerge after the IgM and usually reach a peak level after ∼3 months [41]. As CMV-specific IgM antibodies can also be detected during the reactivation of a latent infection, an IgG avidity test can be applied to distinguish between acute and recurrent infection [42]. IgG antibodies produced during the acute infection have low binding ability, or avidity. In contrast, in latent infection and reactivation, there is an increased in IgG-binding affinity that results in a higher avidity test [43] (Figure 2).

### CMV reactivation

Reactivation of CMV is mostly associated with conditions that cause high levels of systemic inflammatory cytokines, especially TNF-α and immune dysfunction. The reactivation of CMV in immunocompetent hosts may occur in a random fashion throughout life by triggering immunologic memory that controls CMV replication and disease [44]. Immune response to CMV can be exaggerated during certain settings such as sepsis and prolonged hospital stay. A CMV reactivation rate of 31% was observed in a meta-analysis of 18 studies comprising non-immunocompromised individuals with positive CMV IgG antibody at Intensive Care Unit admission [45]. Similarly, because of the defected CMV-specific-cell-mediated immunity, reactivation has also been noted in pregnant patients [46].

Loss of CMV-specific CD4+ and CD8+ T-cells in immunocompromised hosts can lead to a high level of viral replication and fulminant disease [47]. CMV reactivation can be severe among patients with HIV/AIDS, solid-organ transplant, hematopoietic stem-cell transplant, and malignancy with use of immunosuppressive therapy [48, 49]. In the HIV/AIDS setting, CMV reactivation most commonly presents as retinitis but it also involves other organ systems [50]. According to a large cross-sectional study in developing countries, the CMV reactivation rates among patients with AIDS ranged from 3% to 36.6% [51–54]. CMV infection is associated with major morbidity and mortality in patients undergoing organ transplants. The gastrointestinal tract accounts for ∼70% of the CMV-induced tissue-invasive disease among the solid-organ transplant recipients. CMV also has the propensity to infect the transplanted allograft and can cause hepatitis, pneumonitis, myocarditis, pancreatitis, and nephritis [55–57]. The incidence of CMV in solid-organ transplant differs by the serostatus of the donor and recipient, the type of organ transplant, and the preemptive treatment strategies [58].

## CMV and HBV co-infection

There are limited published studies on the association between latent CMV and HBV infection. As both CMV and HBV are prevalent and have the potential to persist and reactivate when the host cellular immunity decreases, it is important to understand the co-infection of these two viruses and their impact on the host. The association between CMV and HBV was studied by Liu *et al*. [59] on 117 patients who received allo-hematopoietic stem-cell transplantation (allo-HSCT). Before the transplantation, 96.4% of the recipients and 92.9% of the donors were seropositive for CMV IgG antibodies, and 11.1% of the donors and 13.7% of the recipients were positive for HBsAg. After allo-HSCT, the incidence of CMV infection, defined as two consecutive positive results for CMV PCR or CMV pp65 antigenemia assay, was 45.3%. The incidence of CMV disease, defined as a combination of clinical symptoms or signs of end-organ disease and presence of CMV infection in a tissue biopsy specimen, was 6.8%. The underlying HBV infection of either donors or recipients did not appear to influence the incidence of active CMV infection. Compared with other patients, patients co-infected with CMV and HBV did not have any overall survival disadvantage at Day 100 (log-rank, *P *=* *0.64). Moreover, no acute exacerbation of hepatitis occurred during the follow-up period. The main limitation of this study is the uncertainty of whether the CMV infection or CMV disease was due to new infection or reactivation of the latent infection [59].

Bayram *et al*. [60] evaluated the hepatic histological patterns of CMV–HBV and CMV–HCV coinfections by measuring the CMV DNA levels and histologic activity scores from liver biopsy samples of antiviral treatment naive patients with CHB (*n *=* *44) and chronic hepatitis C (CHC) (*n *=* *25) and comparing them with control (CMV-positive patients with liver malignancies without hepatitis viruses, *n *=* *36). Anti-CMV IgG antibodies were detected in 86.4% of CHB, 84% of CHC, and 80.5% of the control. None of the patients was anti-CMV IgM-positive. CMV DNA was detected in 52.3% of CHB biopsy samples compared with 13.9% of the control samples (*P *<* *0.001). An increase in necroinflammation and fibrosis scores was noted in CMV-positive CHB compared with CMV-negative CHB patients but the difference was not significant (*P *=* *0.209). CMV–HBV co-infection had lower levels of ALT (115 vs 121 IU/L) and intrahepatic HBV DNA (2.3* *×* *10^7^ vs 3.9* *×* *10^7^ copies/cell) compared with CHB without CMV infection; however, these trends did not reach statistical difference. The authors suggested that the lower HBV DNA levels could be due to CMV-mediated inflammatory response. A similar inhibitory phenomenon of CMV on HBV replication was also noted by Hu *et al*. in a study from China [61]. These clinical observations were in accordance with the *in vitro* findings of Cavanaugh *et al*. [62], in which local induction of cytokines associated with murine cytomegalovirus hepatitis inhibited the replication of HBV and gene expression. Due to the small sample sizes of these clinical studies, the inhibitory effects of CMV on HBV replication and the pathogenesis of HBV CMV co-infection need to be carefully validated.

A possible mechanism of liver injury by CMV infection was proposed by Kasprzak and coworkers [63]. CMV infection can cause increased expression of TNF-alpha and IL-1alpha in pancreatic beta-cells, hepatic macrophages, liver sinusoids, and alveolar macrophages causing acute hepatitis and other organ injuries. Since these cytokines can be expressed by both HBV and CMV, it is difficult to assess the degree of injury contributed by each virus in the setting of co-infection or superimposed infection, especially the viruses that can interact with each other.

## Conclusions

**Figure 3.**
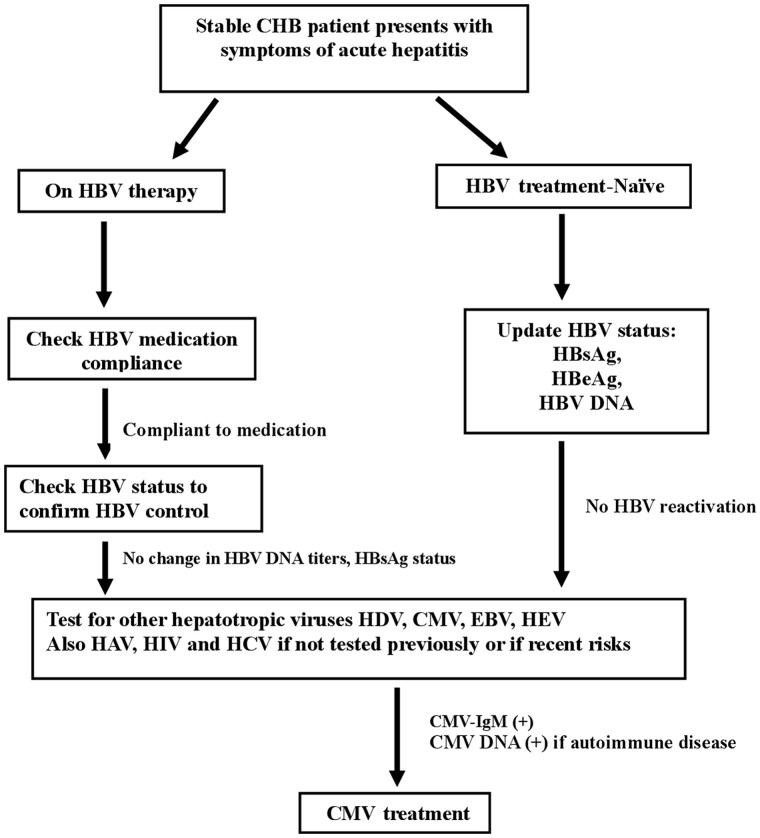
Algorithmic approach to stable CHB patients who present with acute hepatitis. CHB, chronic hepatitis B; HBV, hepatitis B virus; HDV, hepatitis D virus; CMV, cytomegalovirus; EBV, Epstein-Barr virus; HEV, hepatitis E virus; HAV, hepatitis A virus; HIV, human immunodeficiency virus.

HBV and CMV are both hepatotropic yet silent viruses that can cause mild to severe liver disease depending on the immune system of the host. Physicians need to be alert to the possibilities of acute CMV infection or reactivation in a stable chronic hepatitis B patient with hepatitis flare. Standardized guidelines or recommendations need to be established regarding the evaluation and management of patients with HBV CMV co-infection. Based on our experiences, we provided an algorithmic approach to evaluate patients with chronic hepatitis B who present with acute symptoms of hepatitis (Figure 3). Although both HBV and CMV are prevalent, there is scarcity in the literature about the pathogenesis of the co-infection. Moreover, studies reported conflicting results on the inhibitory effect of CMV on HBV replication. Future research initiatives dedicated at understanding the interactions of the CMV and HBV at the molecular level and their combined impact on the overall liver injury are urgently needed.

## Authors’ Contributions

M.A.C. provided the key literature and contributed to manuscript writing and editing; D.T.-Y.L. provided the concept and direction of the review, the learning points, and the editing of the manuscript; M.M.K., M.J.A., and H.H. contributed to the addition of literature, revision, and editing of the final manuscript. M.H.M. and I.A. reviewed the literature and wrote the first manuscript draft; M.M.K. and J.E.G.A. contributed to manuscript editing and graphics. All authors read and confirmed the final version of this paper.

## Conflict of Interest

None declared.
